# Unusual Lower Lip Swelling: A Rare Case of Lip Schwannoma

**DOI:** 10.7759/cureus.19242

**Published:** 2021-11-03

**Authors:** Mohamad Bakir, Joud Enabi, Hanan Almeshal

**Affiliations:** 1 Medicine and Surgery, College of Medicine, Alfaisal University, Riyadh, SAU; 2 Department of Dermatology, Prince Mohammed Bin Abdulaziz Hospital, Ministry of Health, Riyadh, SAU

**Keywords:** case report, verocay bodies, lower lip, oral neural neoplasms, neurofibroma, neurilemmoma, schwannoma

## Abstract

Schwannoma (neurilemmoma) is a benign neoplasm that arises from the nerve sheath's Schwann cells. Between 25% and 40% of all schwannomas are discovered in the soft tissues of the head and neck area, but they are infrequently detected in the oral cavity, with the lips being the most unusual site of involvement. Peripheral nerves in the intraoral cavity originate only 1% of schwannomas despite the fact that lips and oral cavity are heavily innervated anatomical areas. Schwannomas are more common in people between the third and fifth decades of life, and there is no predilection based on gender or race. Here, we report a case of lip schwannomas in a 22-year-old female. The lesion was affecting her lower lip and growing steadily for the past two years. The mass measured 1.5 x 1 cm, involving the lower lip with surface telangiectasia. The patient underwent surgical removal of the lower lip mass, and the mass was sent for histopathological correlation that showed completely excised encapsulated schwannoma with free margins. The patient did not have any postoperative complications and was discharged home on the same day. The patient was followed up in the outpatient clinics, and she made full recovery and was pleased with the outcome.

## Introduction

Schwannomas (also known as neuromas, neurilemmomas, and neurinomas of Verocay) are slow-growing, benign nerve sheath tumors derived from the neural crest and made up entirely of Schwann cells [1,2]. Almost 90% of all schwannomas are sporadic. However, schwannomas may have a genetic basis in sporadic cases and when associated with particular syndromes such as neurofibromatosis type 2, Carney complex, and schwannomatosis [3]. Schwannomas are more common in people between the third and fifth decades of life, and there is no predilection based on gender or race [3]. Schwannomas are most commonly found in the upper limbs, followed by the head, trunk, and lower extremity flexor surfaces. Schwannomas can also be found in the posterior mediastinum, thyroid, spinal roots, bone, retroperitoneum, adrenal glands, gastrointestinal tract, liver pancreas, and lymph nodes [3]. Although 25%-40% of all schwannomas are discovered in the soft tissues of the head and neck area, they are infrequently detected in the oral cavity, with the lips being the most unusual site of involvement, and as a result, lip schwannomas are frequently overlooked in the differential diagnosis [4]. The case of a 22-year-old female patient with schwannoma in the lower lip mucosa that had been growing steadily for the past two years is presented here.

## Case presentation

**Figure 1:**
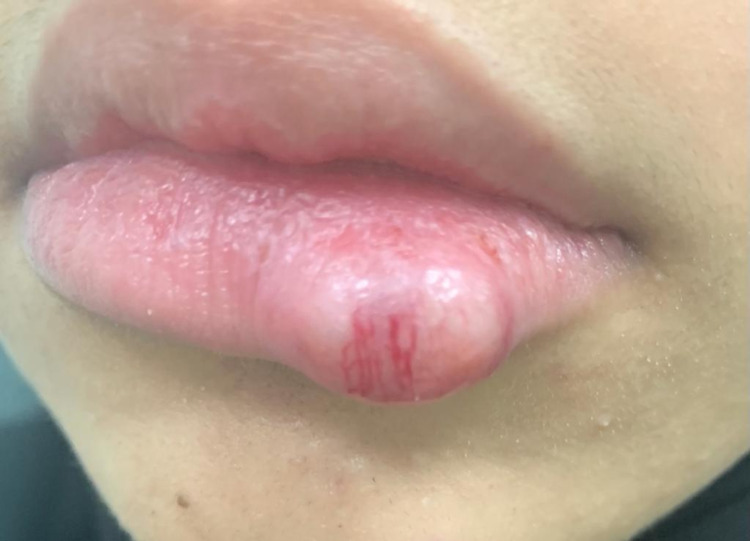
Preoperative image showing single skin-colored firm lower lip mass with surface telangiectasia

**Figure 2:**
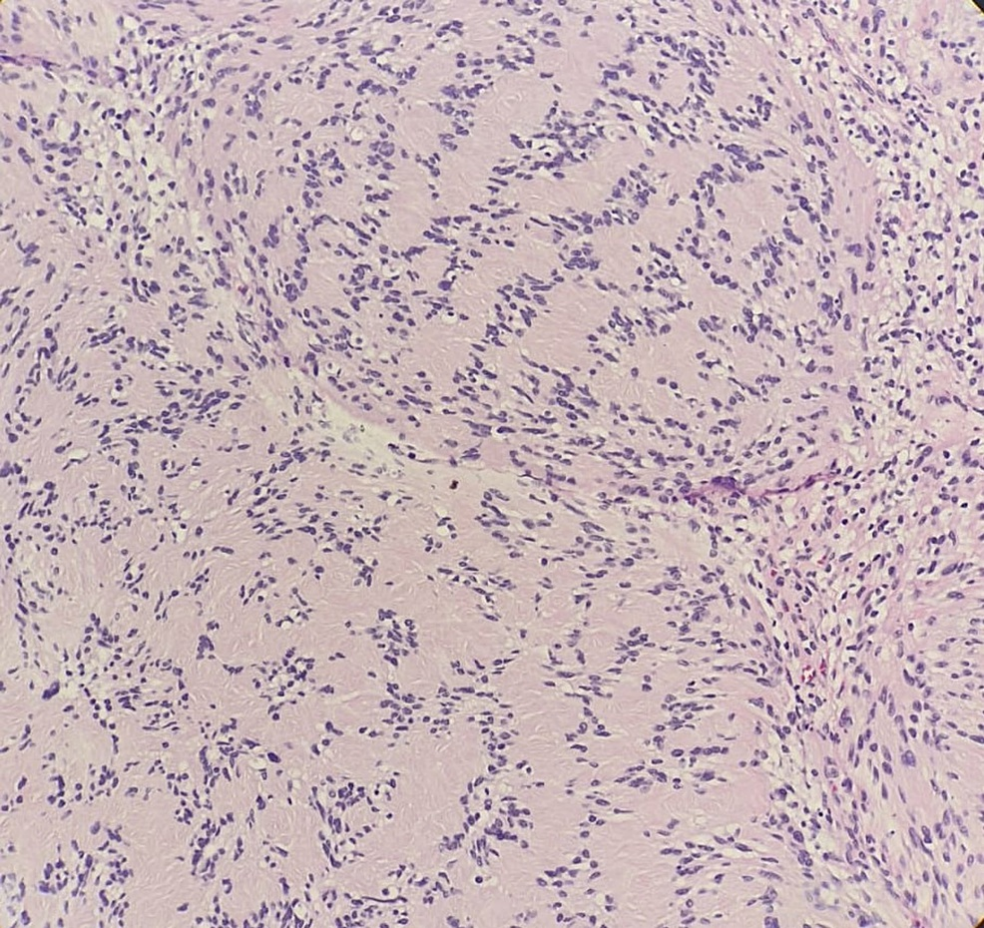
Histopathology slide showing biphasic compact hypercellular Antoni A areas and myxoid hypocellular Antoni B areas with nuclear palisading around the fibrillary process (Verocay bodies)

**Figure 3:**
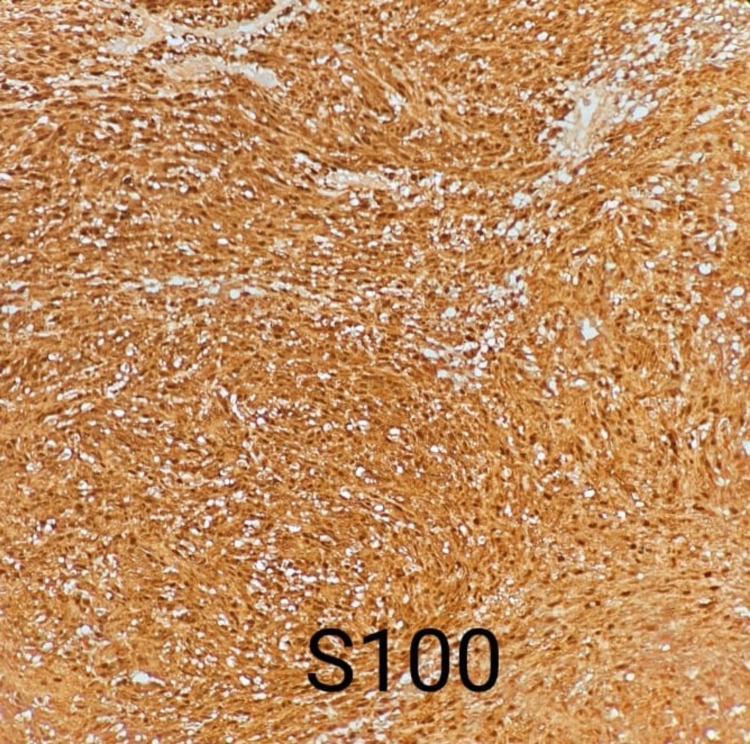
Histopathology slide showing diffuse S100 staining consistent with schwannoma

**Figure 4:**
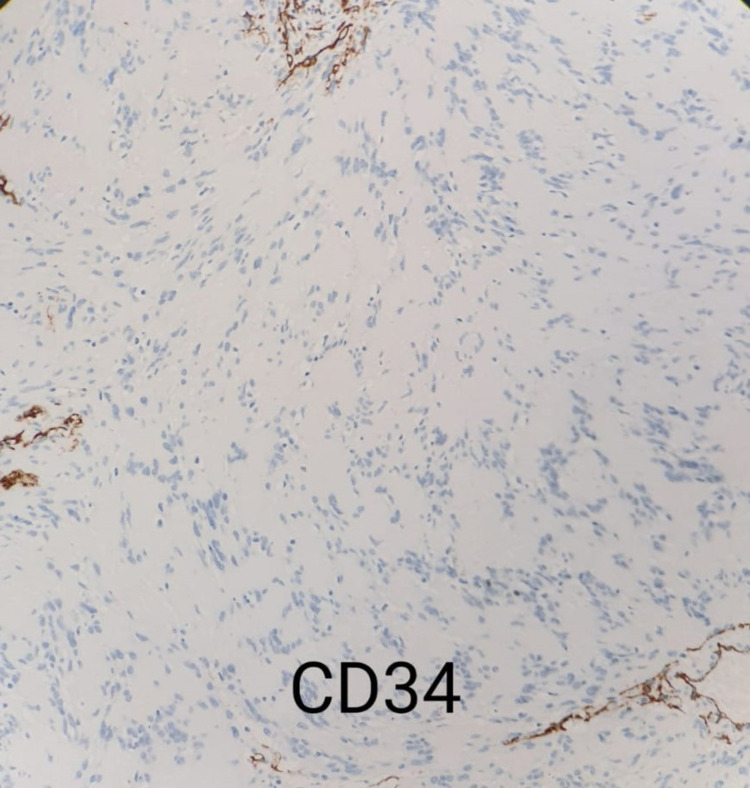
Histopathology slide showing negative cluster of differentiation 34 (CD34) stain (positive in the blood vessels internal control)

**Figure 5:**
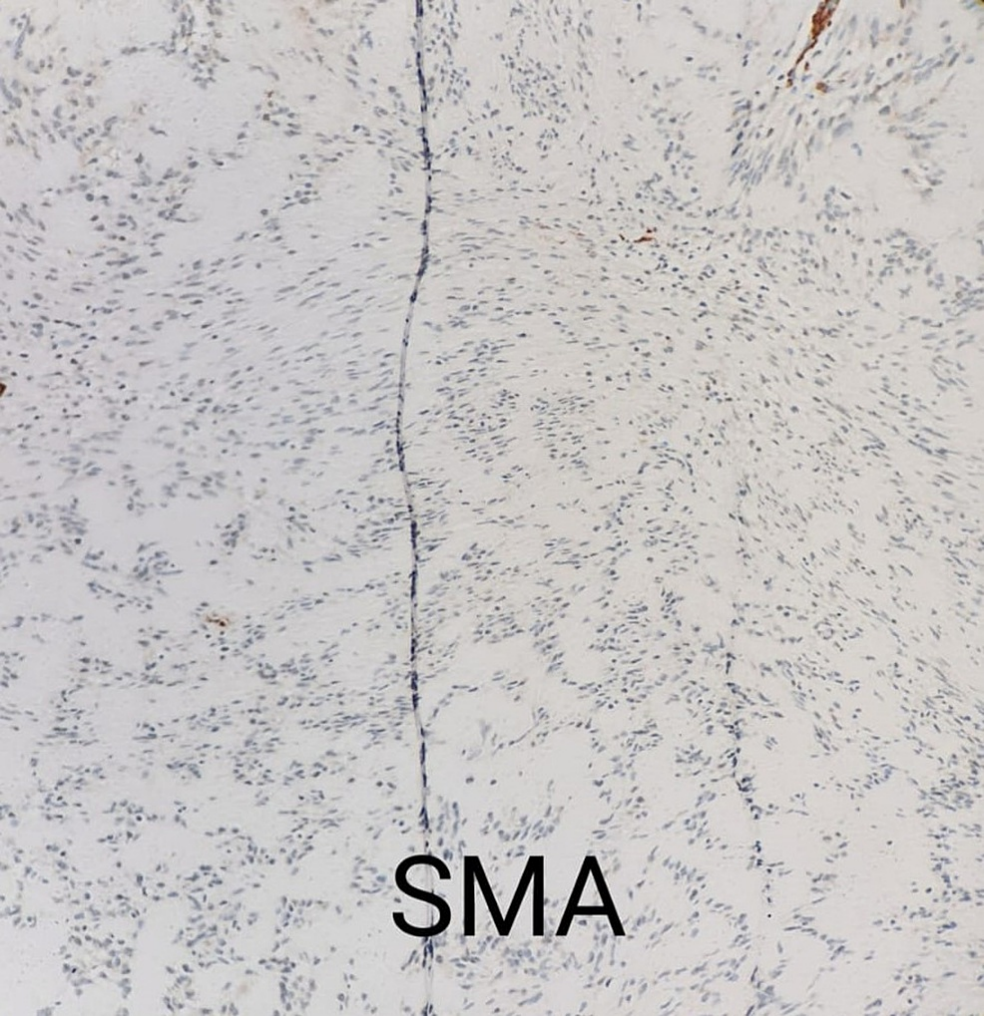
Histopathology slide showing negative smooth muscle actin (SMA) stain (positive in the blood vessels internal control)

**Figure 6:**
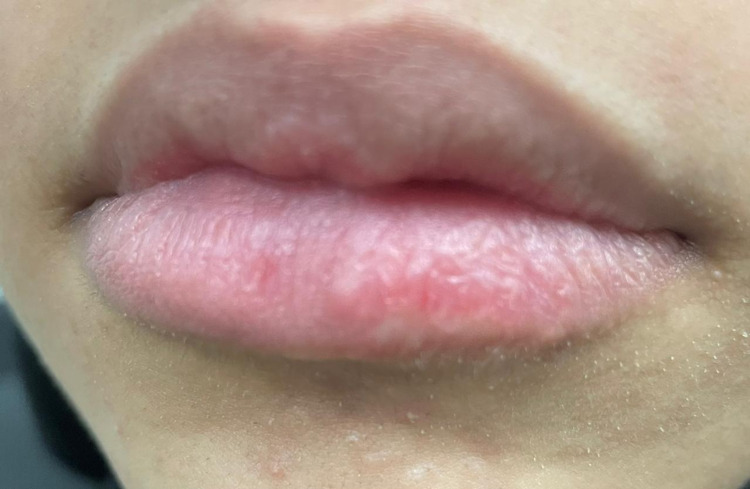
Postoperative image showing the lower lip after full removal of the tumor

A 22-year-old female presented to the dermatology clinic with a history of an asymptomatic skin lesion affecting the lower lip. The lesion started almost two years prior, growing gradually in size. There was no history of discharge or previous ulceration of the lesion. Upon physical examination, the patient was vitally stable with a single, non-tender, well-defined, firm erythematous mass measuring 1.5 x 1 cm, involving the lower lip with surface telangiectasia (Figure *1*). On ultrasound examination, the lower lip showed a well-defined, rounded, hypoechoic soft tissue lesion measuring 1.4 x 0.9 cm, along with mild internal vascularity. Surgical excision with histopathological correlation was recommended. The patient was referred to plastic surgery for further management, and they planned to remove it surgically. Therefore, the patient underwent surgical removal of the lower lip mass under general anesthesia. The tumor was fully excised using a fusiform incision, and dissection around the mass was done. The mass was sent for histopathological correlation, which showed biphasic compact hypercellular Antoni A areas and myxoid hypocellular Antoni B areas with nuclear palisading around the fibrillary process (Figure *2*) with benign free margins. The S100 stain was positive and showed strong and diffuse staining (Figure *3*), the cluster of differentiation 34 (CD34) stain was negative (Figure* 4*), and smooth muscle actin (SMA) was also negative (Figure *5*), which confirmed the diagnosis of schwannoma. The patient did not have any postoperative complications and was discharged home on the same day. The patient was advised to gently rub the lower lip using Vaseline and to start massaging her lower lip two weeks after the operation. The patient was followed up in the plastic surgery and dermatology outpatient clinics, and she made full recovery and was satisfied with the end result (Figure* 6*).

## Discussion

Schwannomas are slow-growing, benign neoplasms that arise from the Schwann cells that surround the sheaths of the peripheral, cranial, or autonomic nerves [5]. Schwannomas grow slowly and might reside for years without causing any symptoms, and may grow to a significant size before becoming symptomatic if they are located in deep-seated structures such as the retroperitoneum [6]. Although the origin is mainly unclear, various causal factors have been proposed, including a rare genetic susceptibility, spontaneous development, external damage, and chronic inflammation [7]. Despite the fact that the lips and the oral cavity are heavily innervated anatomical areas, only 1% of schwannomas originate from the peripheral nerves in the intraoral cavity [4]. Schwannomas can present in a number of locations, which explains why clinical manifestations vary. Tenderness is felt while palpating the lump, and there is a five-year lag between the onset of symptoms and the diagnosis [3]. In histopathological bases, classical schwannoma is an encapsulated tumor made of two different histopathological areas. Antoni A tissue has hypercellular spindle cells, which occasionally palisade surrounding eosinophilic regions (Verocay bodies), and it is positive for S100 protein immunostain. Antoni B tissue has a hypocellular appearance with loose connective tissue in the background [3,8]. Schwannomas are generally solitary, smooth, freely mobile, slow-growing, and minimally invasive tumors; therefore, full surgical excision is the mainstay of treatment to ensure minimal physical disfigurement and recurrence [9,10]. Perioral schwannomas are often associated with unpleasant symptoms such as dysphagia, dysarthria, restricted mouth breathing, snoring, speech and swallowing interference, and in rare cases paresthesia and pain. Furthermore, schwannomas of the lip produce apparent visual deformity for the patient and induce mental discomfort [11,12]. Because this tumor is uncommon in the lips, a diagnosis of lip schwannoma is sometimes ignored until it has progressed to more advanced stages of growth and cosmetic disfigurement [9,13]. Because of the delicate neuronal anatomy within the vast neuronal innervation of the lips, surgical removal of schwannomas in the lips is intrinsically difficult, depending on the size and exact location of the lesion, and even minor injury during excision can result in considerable morbidities such as impaired speech, dysarthria, aspiration, paresthesia, and dysphagia [14-16]. Due to the sheer rarity of this neoplasm and the risks associated with total excision, the ultimate aims for successful care of this tumor are early clinical detection and treatment initiation [17]. In this paper, we have documented an uncommon presentation of schwannoma in the lower lip and suggested that individuals with this tumor have great outcomes if it is detected early and treated immediately.

## Conclusions

Schwannomas are nerve sheath tumors that are formed solely of Schwann cells produced from the neural crest. They are benign, well-encapsulated, and slow-growing tumors. Lip schwannomas are a rare tumor with a high success rate if detected and treated early. For that reason, lip schwannoma must be considered in the initial differential diagnosis while examining a patient with asymptomatic lip growth. In cases of benign lip schwannoma, surgical excision is the gold standard of treatment, with excellent postoperative prognosis and rare incidences of recurrence.
